# Dexmedetomidine vs. haloperidol in delirious, agitated, intubated patients: a randomised open-label trial

**DOI:** 10.1186/cc7890

**Published:** 2009-05-19

**Authors:** Michael C Reade, Kim O'Sullivan, Samantha Bates, Donna Goldsmith, William RSTJ Ainslie, Rinaldo Bellomo

**Affiliations:** 1 Department of Intensive Care Medicine, Austin Hospital and the University of Melbourne, 145 Studley Road, Heidelberg, Victoria, 3084, Australia

## Abstract

**Introduction:**

Agitated delirium is common in patients undergoing mechanical ventilation, and is often treated with haloperidol despite concerns about safety and efficacy. Use of conventional sedatives to control agitation can preclude extubation. Dexmedetomidine, a novel sedative and anxiolytic agent, may have particular utility in these patients. We sought to compare the efficacy of haloperidol and dexmedetomidine in facilitating extubation.

**Methods:**

We conducted a randomised, open-label, parallel-groups pilot trial in the medical and surgical intensive care unit of a university hospital. Twenty patients undergoing mechanical ventilation in whom extubation was not possible solely because of agitated delirium were randomised to receive an infusion of either haloperidol 0.5 to 2 mg/hour or dexmedetomidine 0.2 to 0.7 μg/kg/hr, with or without loading doses of 2.5 mg haloperidol or 1 μg/kg dexmedetomidine, according to clinician preference.

**Results:**

Dexmedetomidine significantly shortened median time to extubation from 42.5 (IQR 23.2 to 117.8) to 19.9 (IQR 7.3 to 24) hours (*P *= 0.016). Dexmedetomidine significantly decreased ICU length of stay, from 6.5 (IQR 4 to 9) to 1.5 (IQR 1 to 3) days (*P *= 0.004) after study drug commencement. Of patients who required ongoing propofol sedation, the proportion of time propofol was required was halved in those who received dexmedetomidine (79.5% (95% CI 61.8 to 97.2%) vs. 41.2% (95% CI 0 to 88.1%) of the time intubated; *P *= 0.05). No patients were reintubated; three receiving haloperidol could not be successfully extubated and underwent tracheostomy. One patient prematurely discontinued haloperidol due to QTc interval prolongation.

**Conclusions:**

In this preliminary pilot study, we found dexmedetomidine a promising agent for the treatment of ICU-associated delirious agitation, and we suggest this warrants further testing in a definitive double-blind multi-centre trial.

**Trial registration:**

Clinicaltrials.gov NCT00505804

## Introduction

Up to 71% of critically ill patients have delirium or psychomotor agitation at some point in their intensive care unit (ICU) stay [1]. Delirium is unpleasant for the patient, and is independently associated with longer hospital stay and six-month mortality [2]. Delirium, along with physiological disturbances (hypoxaemia, hypoglycaemia, drug withdrawal, etc) and pain, often causes psychomotor agitation [3]. Agitation in intensive care is problematic, associated with self-extubation, removal of vascular catheters, increased oxygen consumption and failure to cooperate with treatment [4].

In the early stages of a patient's intensive care treatment, delirium and agitation are often masked using analgesics and sedatives. However, patients may remain delirious and agitated after their underlying illness has resolved, when they are otherwise suitable for extubation. Despite little published evidence of efficacy, haloperidol, a centrally acting dopamine antagonist also used in the treatment of major psychoses, is the drug recommended and most commonly prescribed for this indication [3]. Haloperidol has a number of side effects, including extrapyramidal reactions and (rarely) neuroleptic malignant syndrome, although these may be due to a first-pass metabolite [5], and so are less relevant with the intravenous route. The most problematic adverse effect in the ICU is prolongation of the corrected QT (QTc) interval [6], which can precipitate fatal arrhythmias [7,8].

The ideal treatment for ICU-associated delirious agitation would relieve symptoms without causing excessive sedation, have fewer side effects than haloperidol, have little interaction with other drugs and would be easily titrated. Analgesia could reduce opioid use, also lessening delirium. Dexmedetomidine, a selective α_2 _agonist, has all of these properties [7,9]. One case series reported the successful use of dexmedetomidine in this context [10], but there have been no controlled trials of dexmedetomidine for the treatment, as opposed to prophylaxis [11-13], of ICU-associated delirious agitation. We hypothesised that dexmedetomidine would be more effective than haloperidol in the treatment of ICU-associated delirious agitation in mechanically ventilated patients. We report the results of our pilot study assessing the feasibility of trial design and the safety of both haloperidol and dexemedetomidine.

## Materials and methods

### Patients

We studied patients in our 20-bed general medical/surgical ICU, which admits approximately 2000 patients a year, of whom 50% undergo mechanical ventilation. The median Acute Physiology and Chronic Health Evaluation (APACHE) III score is 48 (interquartile range (IQR) 34 to 65), mean length of stay is 2.8 days and mortality is 13%, which is typical of a large Australian academic ICU [14]. From April 2006 to August 2008 we asked clinicians to identify patients who they considered required mechanical ventilation only because their degree of agitation (e.g. Richmond Agitation Sedation Scale (RASS) [15] score ≥ 2) required such a high dose of sedative medication that extubation was not possible.

Patients were excluded if they could not be extubated even if their agitation were corrected: for example, those receiving high-dose opioid analgesia for pain, those with a plan to shortly return to the operating theatre, those likely to require ongoing airway protection or ventilatory support, and those who remained so physiologically unstable that extubation would be unsafe. Patients were also excluded if they had had an adverse reaction to haloperidol or α_2 _agonists. Patients who met the inclusion criteria were, by virtue of their delirium, unable to give informed consent. In all cases, following the assent of the patients' next of kin, application was made to the Victorian Civil and Administrative Tribunal, who as the patients' temporary legal guardian, gave consent to their enrolment. This is the mandatory procedure in the state of Victoria for the involvement in clinical research of patients unable to give consent. The study protocol was approved by the Austin Hospital Human Research Ethics Committee and registered with the US Government Clinical Trials Registry (NCT00505804).

### Study intervention

Eligible patients were allocated to either haloperidol or dexmedetomidine using numbered envelopes into which a card indicating patient allocation had been placed according to a computer-generated random-number sequence. Dexmedetomidine was administered intravenously as a maintenance infusion of 0.2 to 0.7 μg/kg/hour for as long as deemed necessary by the treating physician. The clinician was given the option of using a loading dose of 1.0 μg/kg intravenously over 20 minutes, as recommended by the manufacturer. Haloperidol was administered as a continuous intravenous infusion of 0.5 to 2 mg/hour for as long as necessary, preceded by a loading dose of 2.5 mg if desired.

With continuous assessment and in consultation with the treating physician, bedside nursing staff adjusted drug infusion rates as necessary (re-assessing at least every four hours), aiming to minimise psychomotor agitation and achieve a RASS score of 0. No rigid protocol governed the titration of each infusion within the limits defined. Clinical personnel were not blinded to the study drug. Treatment was continued for as long as clinically indicated, including following extubation if required, unless any adverse effect developed that necessitated drug discontinuation. As dexmedetomdine was not on our hospital formulary, once it had been stopped it could not be restarted; haloperidol could be continued (by infusion or bolus) without restriction.

### Intercurrent care

No other element of patient care was affected by the trial. Clinicians were free to prescribe any sedative or anxiolytic medication other than dexmedetomidine or haloperidol, and all such medication use was recorded. Our unit has no strict protocol for the use of sedatives in intubated patients, although patients expected to be soon weaned from mechanical ventilation are generally prescribed propofol, while others are given midazolam. Intravenous lorazepam is not available in Australia. Similarly, our unit has no formal protocol for weaning from mechanical ventilation: the bedside nurse is responsible for transitioning the patient from mandatory to spontaneous ventilation as soon as possible, with frequent (< every four hours) assessment. The decision to extubate can occur at any time of day or night. During the trial, the timing of tracheostomy was at the discretion of the treating clinician, based on the clinical impression that the patient would be likely to require prolonged mechanical ventilation; however, again, no objective criteria were used.

### Data collection

Upon enrolment, baseline data collected included demographic characteristics, diagnosis, APACHE II score and the use of physical restraint and sedative medication in the preceding 24 hours. During study drug infusion, clinical data were recorded by the bedside nurses as representative values for each four-hour period. Data collected included study drug rate, use of other sedatives, RASS score, Intensive Care Delirium Screening Checklist (ICDSC) score [16], requirement for physical restraint, mean arterial pressure, requirement and rate of vasopressors and inotropes, and the presence of arrhythmias or any other adverse event. The QTc interval was assessed every eight hours. Clinical data were collected until the study drug was discontinued, and outcomes sought until hospital discharge.

### Endpoints

The primary endpoint was time from the commencement of study drug to extubation. In the primary analysis, patients who underwent tracheostomy were analysed as having been extubated at that point (see discussion for rationale), but in a supplementary analysis this was also treated as censored data. Secondary efficacy endpoints included time from commencement of study drug to ICU discharge, time taken to achieve a satisfactory sedation score, and the need for supplemental sedative and analgesic medication. Secondary safety endpoints included the change in QTc interval, the duration and rate of vasopressor or inotropic support, and the requirement for re-intubation.

### Statistical analysis

Using time to extubation as the primary outcome measure, and assuming that the mean ± standard deviation time to extubation in these agitated patients was 72 ± 20 hours, we calculated a study of 20 patients would have an 80% power of detecting a difference in time to extubation of 24 hours in the treatment group with a certainty of 95%. Categorical baseline and outcome data were compared using chi-squared tests, while continuous data was assessed graphically and compared using Mann-Whitney U tests or Student's t tests as required. Univariate survival analysis of time to extubation was performed using the log-rank test, and a Cox proportional hazards model of time to extubation was constructed using backward elimination, with the initial model incorporating all listed baseline data and the final model being that which produced the best fit. All statistical calculations were performed using Stata version 9.2 (StataCorp, College Station, Texas, USA).

## Results

**Figure 1:**
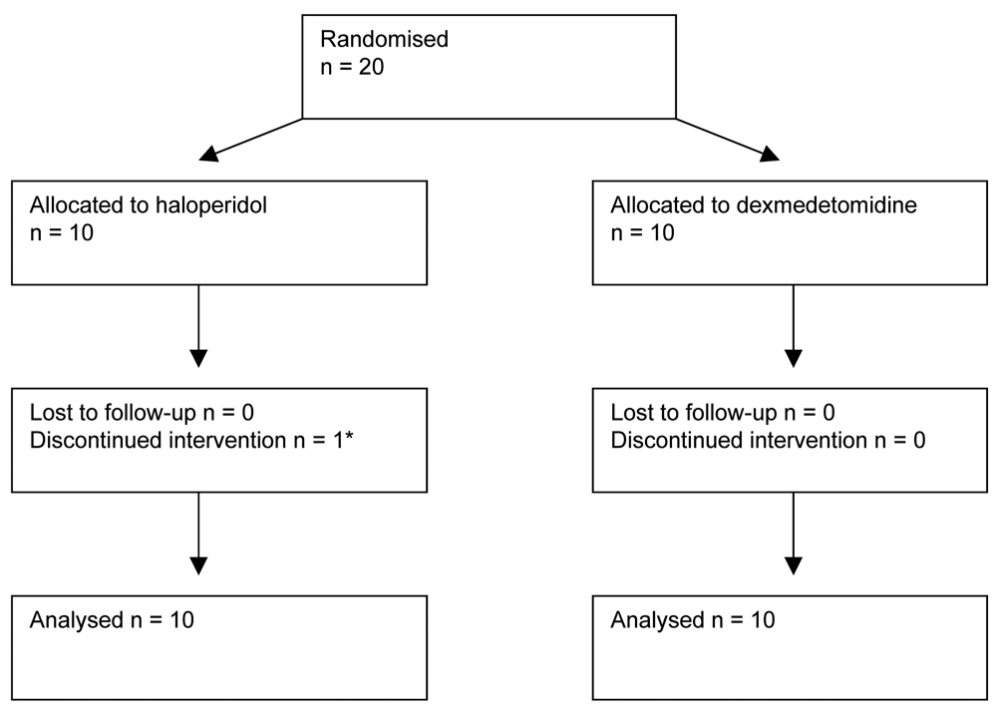
[CONSORT patient flow diagram 44]. * Intervention was discontinued because of consultant physician concern at the length of the QTc interval.

Twenty patients were recruited, with 10 allocated to dexmedetomidine and 10 to haloperidol (Figure 1). No eligible patients' relatives refused consent, and no patients were lost to follow-up. There were no significant differences in the baseline characteristics of the treatment groups (Table 1). Only three patients were female. Eight patients received a bolus of dexmedetomidine, and six a bolus of haloperidol (Table 2). Patients received the intended infusion rates of their allocated study drug almost all of the time they were intubated. Seven of the patients randomised to dexmedetomidine had the infusion continued after extubation; of those that continued, the median duration was 15 (IQR 1 to 26) hours. Only four patients continued receiving haloperidol after extubation, for 6.5 (IQR 2 to 16.5) hours.

**Table 1:** Baseline patient demographic and clinical characteristics

	Dexemedetomidine	Haloperidol	*P*
N	10	10	
Age, years: median (IQR)	52 (42 to 69)	68.5 (43 to 78)	0.241
Males: %	90	80	0.531
APACHE II score in the 24 hours immediately prior to enrolment: median (IQR)	13.3 (10 to 18)	15.5 (11 to 19)	0.383
Physical restraint prior to enrolment: %	80	50	0.160
Midazolam use: %	60	40	0.371
Propofol use: %	70	70	1.000
Haloperidol use: %	30	10	0.264
Morphine use: %	80	80	1.000
Other sedative or anti-psychotic use: %	0	0	
Time intubated prior to randomisation, hours: median (IQR)	45.0 (34.5 to 73.3)	65.2 (28.0 to 87.0)	0.496
RASS -2 to 1 (ie. desired level of sedation and agitation control) at enrolment: %	30	10	0.264
ICDSC ≥ 4 (ie. delirium present) at enrolment: %	30	40	0.405
ICDSC ≥ 4 at any stage prior to or during infusion of trial drug: %	50	50	1.00
ICDSC > 0 (ie. at least subsyndromal delirium present) at enrolment: %	80	100	0.136
ICDSC > 0 at any stage prior to or during infusion of trial drug: %	100	100	1.00
Surgical diagnosis: %	70	30	0.074
Admission diagnosis			0.493
Pneumonia, %	0	20	
Other sepsis, %	20	20	
Post cardiothoracic surgery, %	30	10	
Post neurosurgery, %	30	20	
Other, %	20	30	

APACHE = Acute Physiology and Chronic Health Evaluation; ICDSC = Intensive Care Delirium Screening Checklist; IQR = interquartile range; RASS = Richmond Agitation Sedation Scale.

**Table 2:** Interventions

	Dexemedetomidine	Haloperidol	*P*
Time receiving study drug infusion while intubated, %: median (IQR)	100 (99.1 to 100.0)	94.26 (68.9 to 100.0)	0.2755
Loading dose given, %	80	60	0.329
Drug rate of infusion during the periods when it was administered: mean (95% CI)	0.47 (0.33 to 0.62) μg/kg/hour	1.43 (0.96 to 1.90) mg/hour	N/A
Study drug continued after extubation, %	70	40	0.18
Time study drug continued after extubation, hours: median (IQR)	2.5 (0.0 to 26.0)	0.0 (0.0 to 2.0)	0.15
Of patients who continued study drug after extubation, time continued, hours: median (IQR)	15 (1 to 26)	6.5 (2 to 16.5)	0.57

CI = confidence interval; IQR = interquartile range.

### Primary endpoint

**Figure 2:**
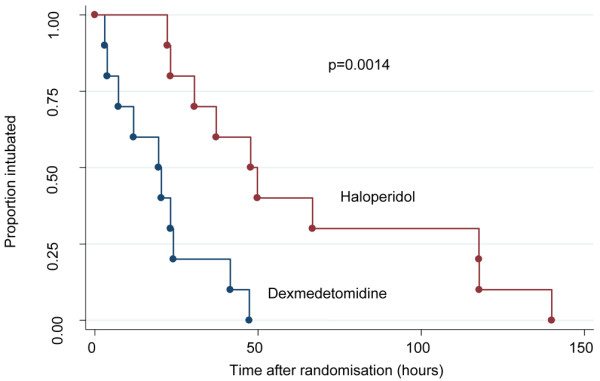
**Graph showing time to extubation**.

Following commencement of the study drug, patients randomised to dexmedetomidine were extubated significantly sooner than those receiving haloperidol (19.9 (IQR 7.3 to 24.0) hours vs. 42.5 (IQR 23.2 to 117.8) hours, *P *= 0.016; Table 3). Three patients randomised to haloperidol eventually underwent tracheostomy at 31, 48 and 140 hours after randomisation. When these patients were excluded from the analysis, the difference in time to extubation remained significant (dexmedetomidine 19.9 (IQR 7.3 to 24) hours vs. haloperidol 49.8 (IQR 23.2 to 117.8) hours; *P *= 0.0147). Time to extubation was also significantly shorter for patients receiving dexmedetomidine in a univariate survival analysis (Figure 2); this conclusion remained unchanged when patients undergoing tracheostomy were censored (log rank test, n = 10 and 7, *P *= 0.009). The best-fit survival model adjusting for baseline differences found older age and having been on midazolam, propofol or haloperidol prior to randomisation all significantly (*P *< 0.05) reduced the likelihood of earlier extubation. Having been restrained prior to randomisation and a higher APACHE II score on entry all increased the chance of early extubation. After adjustment for all these factors, randomisation to dexmedetomidine remained the strongest and most statistically significant (*P *= 0.001) predictor of early extubation.

**Table 3:** Results: efficacy

	Dexmedetomidine	Haloperidol	*P*
*Primary*			
Time to extubation, hours: median (IQR)	19.9 (7.3 to 24.0)	42.2 (23.2 to 117.8)	0.016

*Secondary*			
Time to ICU discharge after randomisation, days: median (IQR)	1.5 (1 to 3)	6.5 (4 to 9)	0.0039
Total ICU length of stay, days: median (IQR)	4.5 (2 to 7)	8.0 (7.0 to 11.0)	0.0093
Time taken to achieve a satisfactory RASS agitation score (-2 to 1), hours: median (IQR)	4 (0 to 7)	18 (9 to 22)	0.071
Time taken to achieve a satisfactory ICDSC score (< 4), hours: median (IQR)	0 (0 to 2)	0 (0 to 2)	0.509
Proportion of time with a satisfactory RASS agitation score (-2 to 1), %: median (IQR)	50.5 (20 to 78)	26.5 (13 to 42)	0.256
Proportion of time with a satisfactory ICDSC score (< 4) when assessable, %: median (IQR)	95.5 (51 to 100)	31.5 (17 to 97)	0.122
Proportion of time with a desirable ICDSC score (< 1) when assessable, %: median (IQR)	61.0 (0.0 to 100.0)	0.0 (0.0 to 0.0)	0.134
Required restraint at any time while on study drug, %	90	80	0.53
Of patients requiring restraint at any time while on study drug, time to first not requiring restraint for > 4 hours, hours: median (IQR)	18 (7.3 to 38.5)	38 (26.3 to 49.8)	0.03
Need for supplemental sedative or analgesic medication, %			
Propofol	60	80	0.33
Midazolam	20	10	0.53
Morphine	30	40	0.64
Of patients requiring supplemental sedative or analgesic medication, dose rate: mean (95% CI)			
Propofol, mg/hour	87.7 (15.5 to 160.0)	123.4 (30.4 to 216.3)	0.504
Midazolam, mg/hour	1.0 (1.0 to 1.0)	2.4 (N/A)	N/A
Morphine, mg/hour	1.0 (0.5 to 1.5)	1.6 (0.3 to 2.8)	0.28
			
Of patients requiring supplemental sedative or analgesic medication, % time this was required: mean (95% CI)			
Propofol	41.2 (0 to 88.2)	79.5 (61.8 to 97.2)	0.05
Midazolam	0 (0 to 0)	0 (N/A)	N/A
Morphine	0 (0 to 0)	32.9 (0 to 100)	0.29
Required tracheostomy	0	3	0.06

CI = confidence interval; ICDSC = Intensive Care Delirium Screening Checklist; ICU = intensive care unit; IQR = interquartile range; RASS = Richmond Agitation Sedation Scale.

### Secondary endpoints: efficacy

Patients who received dexmedetomidine were discharged from the ICU significantly earlier than those randomised to haloperidol (Table 3), and also had a shorter overall ICU length of stay. Dexmedetomidine patients tended to achieve satisfactory sedation scores more quickly, and they tended to spend a greater proportion of time with satisfactory scores. Although all but three patients required mechanical restraint at some point while receiving the study drug, those randomised to dexmedetomidine had this removed significantly earlier. Most patients received supplemental propofol: those randomised to dexmedetomidine required this for a significantly shorter proportion of the time they were intubated (41.2% vs. 79.5%, *P *= 0.05), and at a (non-significantly) lower dose.

### Secondary endpoints: safety

No patients died while in the ICU, but one patient who had received haloperidol died in the general ward from their underlying disease process, unrelated to study medication (Table 4). The mean QTc interval in the two groups was no different prior to study entry, but there was a strong trend towards more patients in the haloperidol group having a prolongation of their QTc interval (compared with baseline) during study drug infusion. There were no significant differences in the rate or duration of norepinephrine required, and only two patients in each group required the institution or a significant increase in the rate of norepinephrine in the eight hours after study drug commenced. Patients who received a dexmedetomidine bolus had no clinically significant hypotension or increased vasopressor requirement. One patient discontinued haloperidol after receiving 9.5 mg over 20 hours, because their consultant physician was concerned at the new onset of atrial fibrillation immediately preceded by new prolongation of their QTc interval to 0.437 seconds. There were no self-extubations, and no patient inadvertently dislodged a central venous catheter. There were no other reported adverse events, and no patients required reintubation.

**Table 4:** Results: safety

	Dexmedetomidine	Haloperidol	P
ICU mortality, n	0	0	1.00
Hospital mortality, n	0	1	0.31
QTc interval prior to study drug, sec: mean (95% CI)	0.411 (0.384 to 0.438)	0.426 (0.395 to 0.457)	0.41
QTc interval while on study drug, sec: mean (95% CI)	0.395 (0.365 to 0.425)	0.446 (0.423 to 0.457)	0.0061
Patients with abnormal QTc interval (> 0.440 sec) while on study drug: %	40	40	1.00
Patients with longer QTc interval than baseline while on study drug: %	30	70	0.07
Arrhythmia while on study drug: %	20	20	1.00
Patients requiring norepinephrine* infusion while on study drug: %	80	50	0.16
Patients newly requiring norepinephrine or a 20% increase in norepinephrine* infusion in the 8 hours after commencement of study drug: %	20	20	1.00
Of patients requiring norepinephrine, proportion of the time while on study drug receiving norepinephrine: mean (95%CI)	59.8 (17.9 to 100.0)	34.4 (0.0 to 87.1)	0.37
Of patients requiring norepinephrine, level of infusion (μg/min) while on study drug: mean (95%CI)	2.51 (0.07 to 4.90)	3.97 (0.00 to 11.07)	0.55
Any adverse event attributed to the study drug: %	0	10**	0.31
Patients requiring reintubation: n, %	0	0	1.00

* norepinephrine was the only inotropic or vasopressor medication used in any study patient

** excessive prolongation of the QTc interval, necessitating drug discontinuation

CI = confidence interval; ICU = intensive care unit; QTc = QT interval corrected for heart rate.

## Discussion

This is the first study to demonstrate that dexmedetomidine is more effective than conventional haloperidol therapy for the treatment of combined agitation and delirium in intubated patients in the ICU. Dexmedetomidine, in comparison to haloperidol, safely shortened the time to extubation, reduced ICU length of stay, hastened liberation from mechanical restraint, reduced the need for supplementary sedation, reduced QTc interval prolongation and possibly reduced the need for tracheostomy.

### Efficacy

In the primary analysis, we treated tracheostomy as equivalent to extubation. We contend this is reasonable as tracheostomy in this context represents the failure of treatment of agitation and delirium, reflecting the clinician's decision that the patient would be unlikely to be soon extubated. Had the three patients in the haloperidol group not undergone tracheostomy, they could only have remained intubated for longer; hence our analysis biases towards observing less difference between the two groups. We nonetheless also analysed the data by excluding these patients and by treating them as censored in the survival analyses; our conclusion was unchanged.

There is a theoretical concern that given its short half-life, when dexmedetomidine is discontinued a patient might return to a state of agitation so severe as to require reintubation. That none of our patients required reintubation does not discount this possibility, given the small number we studied. We continued dexmedetomidine following extubation for as long as the treating clinician felt the patient was at risk of reintubation due to agitation. Had we not done so, this risk may or may not have been manifest.

### Safety

Dexmedetomidine shares no common adverse reactions with haloperidol. Transient hypertension during the administration of the loading dose, followed by hypotension and bradycardia, are the only adverse reactions reported [7]. Our study was not powered to observe anything but marked haemodynamic effects, so we can only conclude that dexmedetomidine did not cause a dramatic increase in vasopressor requirement.

### Rationale for trial design

Dexmedetomidine has been studied and marketed primarily as a sedative alternative to propofol or benzodiazepines. The sedative, analgesic and anxiolytic effects of dexmedetomidine have been convincingly demonstrated [9,17-20]. These trials were performed in the initial postoperative period and so the approved product information limits the duration of dexmedetomidine infusion to 24 hours [7]. However, prolonged infusions have been used successfully in case series and published trials [11-13,21,22]. We considered allowing clinicians to decide when to terminate the infusion would be safer and more effective than imposing an arbitrary time limit.

Dexmedetomidine might prevent agitation by reducing the use of other sedatives known to cause delirium [23]. In a trial involving 106 patients, dexmedetomidine resulted in more days alive without delirium or coma and more time at the targeted level of sedation than did lorazepam [11]. However, concerns were subsequently raised about the equivalence of dosing [24], cost-effectiveness [25] and the validity of the outcome measure [26]. A second trial comparing dexmedetomidine to midazolam as a sedative in 375 patients found dexmedetomidine associated with significantly less delirium and a shorter duration of intubation [13]. However, even if cost-effective in preventing delirium elsewhere [27], widespread application of dexmedetomidine as a sedative is prohibitively expensive in our current context. We therefore wondered whether dexmedetomidine might be effective in the treatment of established delirium, reasoning that this might be sufficiently cost-effective.

Despite widespread use and incorporation into international guidelines [3], there is no evidence from placebo-controlled trials supporting the use of haloperidol (or indeed any other medication) in the management of ICU-associated delirium [28]. Our results may therefore reflect comparison with an ineffective agent. Olanzipine and risperidone are the only other agents used in our management of critical illness delirium: both have been compared with haloperidol; neither is more effective [29,30]. We therefore concluded that, although imperfect, haloperidol represented 'standard care' in our management of delirium in the ICU.

We administered haloperidol by infusion rather than conventional bolus dosing. This approach has been used successfully in case series of ICU patients [31,32] and is presented as theoretically superior in current guidelines [3]. The relatively long half-life of haloperidol (12 to 36 hours) means that control of agitation when the infusion rate is increased may take longer in comparison to dexmedetomidine. This concern probably does not explain our results, as haloperidol tended to be used at the upper end of the permitted dose in most patients for most of the time it was infused. We chose to use haloperidol by infusion for two main reasons. First, we were concerned that 'on demand' boluses of haloperidol might lead to relative underdosing compared with dexmedetomidine by continuous infusion. Second, we designed our trial as a prelude to a larger double-blind study, in which (to preserve blinding) both study drugs would need to be given by continuous infusion. In the absence of evidence, we selected a dose range of haloperidol that reflected our usual practice. Although this was somewhat less than the 3 to 11.35 mg/hour (in a 75 kg patient) recommended by current guidelines [3], a dose of 272 mg haloperidol (as per those guidelines) in a 24-hour period substantially exceeds our routine practice. We nonetheless accept that we may have found haloperidol less effective than dexmedetomidine due to an inadequate dose.

As is the case for haloperidol, the optimal dose rate of dexmedetomidine is also not well characterised. We used up to the maximum dose of dexmedetomidine licensed for use in Australia (and elsewhere) at the time of the study, which was 0.7 μg/kg/hour. Two large randomised controlled trials have now safely used doses up to 1.4 [13] and 1.5 [11] μg/kg/hour: at higher doses dexmedetomidine might be even more effective for this indication.

Our study was not blinded. We were concerned at the potential for QTc interval prolongation with high doses of haloperidol [8], particularly as continuous infusion is not our usual practice. We also noted the risk of hypotension associated with dexmedetomidine [9], which was at the time an unfamiliar drug in our unit. Having not observed significant complications with either drug, we suggest a larger, blinded trial would be sufficiently safe.

### Strengths and limitations

This is a pilot study, with significant limitations. The principal concern is the lack of blinding. If our consultant physicians and bedside nurses had more confidence in dexmedetomidine than haloperidol, they may have been more inclined to attempt earlier extubation in dexmedetomidine patients, or proceed to tracheostomy in patients receiving haloperidol. This is especially true given our usual clinical practice of not using objective criteria to make such decisions, although imposing such restrictive criteria would potentially have led to a significant change in intercurrent care. However, the observed magnitude of the differences between the groups is difficult to attribute to factors other than, at least in part, the different effects of the drugs.

We allowed physicians to decide whether or not to use an initial bolus of dexmedetomidine. There is growing evidence that such a bolus may cause adverse cardiovascular effects (hypotension or hypertension) [22,33] while adding little sedation [21,34]. Insufficient numbers may have precluded observation of such effects. Similarly, we may have studied too few patients to allow us to observe clinically important rebound hypertension and tachycardia associated with the abrupt cessation of dexmedetomidine. However, others have found this quantitatively insignificant [21]. The small size of our study also raises the possibility that our results are confounded by unobserved imbalances in the baseline characteristics of the two groups. Although this cannot be excluded and is inherent to every pilot study, again the magnitude of the effect observed adds plausibility to our findings.

We did not keep a screening log, but as our ICU admits about 1000 mechanically ventilated patients per year, it is conceivable that approximately 2300 patients were informally screened but only 20 enrolled. At the time of the study, we, like most others [35,36], did not routinely assess for delirium using a screening tool. Despite its known high incidence, clinical underdiagnosis of delirium in the ICU [37,38] partly explains our recruiting difficulty. Additionally, we required patients be unsuitable for extubation only because of agitation. Dexmedetomidine may be effective in delirious patients with ongoing physiological instability; indeed in comparison with benzodiazepines others have found this to be the case [11,13]. However, while there are several well-studied and effective sedatives, we were concerned that this was not true for drugs specifically targeting delirious agitation. Although our study reflects use of dexmedetomidine in the context of our routine practice at the time, we propose that any follow-up trial should actively screen for delirium using objective criteria. Additionally, we only studied patients with agitated delirium. Hypoactive delirium may be eight times more common (61%) than delirium associated with agitation (8%) [39], but, while no less important, hypoactive delirium is difficult to identify without active screening. The results of our pilot study do not allow us to comment on the management of hypoactive delirium.

We have no reliable data on pre-morbid cognitive impairment in these patients, the presence of intercurrent conditions known to be associated with delirium or any history of substance abuse. Any imbalance in these factors between the two groups may have confounded the results, in particular as dexemedetomidine may be especially useful for managing drug withdrawal [40,41]. Having identified these potential confounders, we suggest a future definitive trial examine these factors in detail.

By chance, there were more surgical patients in the dexmedetomidine group, although with the small size of the study this difference was not significant. Dexmedetomidine is an analgesic and pain causes agitation, so dexmedetomidine may have appeared more effective because it was a better treatment for pain. However, in multivariate analysis, surgical diagnosis was not a significant predictor of time to extubation, arguing against this hypothesis.

Relatively few (50%) of our patients had delirium, as identified by an ICDSD score of 4 or above. This is surprising, as the impression of their treating clinicians was that each had delirium as the cause of their agitation. However, Ouimet and colleagues [42] demonstrated that 'subsyndromal' delirium (an ICDSC score > 0) was also associated with poor outcome, and all of our patients has an ICDSC score more than 0 at some point, supporting the clinical impression that they were delirious. Although agitation is commonly caused by delirium, this is not always the case; pain and presence of an endotracheal tube alone can be sufficient to cause agitation. Some patients were too deeply sedated at the time of enrolment to permit proper use of the ICDSC. Presumably this sedation had been administered because of earlier agitation, which we were then unable to objectively record. A significant weakness of this pilot study is therefore the lack of objective evidence of delirium in many patients prior to randomisation, a deficiency which should be rectified in any confirmatory trial by the use of active screening using either the ICDSC or the Confusion Assessment Method for the Intensive Care Unit (CAM-ICU) [43].

## Conclusions

Despite its many limitations, confidence in the results of our study is increased by the magnitude of the effect size and by our use of objective, easily quantified outcome measures, which despite the listed concerns would have been difficult to artificially manipulate. Nonetheless, given its small size and unblinded nature, we recommend against using our conclusions to support a widespread change in practice. Our study supports, but does not conclusively demonstrate, the efficacy and safety of dexmedetomidine at its currently licensed dose for longer than 24 hours for this indication. We suggest our results justify the conduct of a larger, blinded randomised controlled trial, incorporating objective entry criteria and active protocolised screening for agitated delirium, allowing use of dexmedetomidine up to 1.5 μg/kg/hour, and incorporating formal cost-effectiveness and quality-of-life analyses and follow-up to 90 days.

## Key messages

• Haloperidol is the drug recommended and most commonly used for the treatment of ICU-associated delirious agitation, but there is little evidence to support this practice.

• Dexmedetomidine is a selective α 2-agonist licensed for use as a postoperative sedative that may have advantages over haloperidol in this context.

• In this pilot study, we randomised 20 patients who remained intubated only because of agitated delirium to receive infusions of either haloperidol or dexmedetomidine in addition to usual care.

• Dexmedetomidine significantly shortened time to extubation and decreased ICU length of stay.

• We suggest dexmedetomidine is a promising agent for this indication, and warrants testing in a multicentre effectiveness trial.

## Abbreviations

APACHE: Acute Physiology and Chronic Health Evaluation; CAM-ICU: Confusion Assessment Method for the Intensive Care Unit; ICDSC: Intensive Care Delirium Screening Checklist; ICU: intensive care unit; IQR: interquartile range; QTc: QT interval corrected for heart rate; RASS: Richmond Agitation Sedation Scale.

## Competing interests

The authors declare that they have no competing interests.

## Authors' contributions

MR conceived and designed the study, analysed the results and drafted the manuscript. KO, SB, DG and WA contributed to the design of the study, recruited patients, and collected and verified data. RB conceived and designed the study, oversaw its conduct and revised the manuscript. All authors read and approved the final manuscript.
